# Hypertension-associated C825T polymorphism impairs the function of Gβ3 to target GRK2 ubiquitination

**DOI:** 10.1038/celldisc.2016.5

**Published:** 2016-04-26

**Authors:** Zhengyu Zha, Xiao-Ran Han, Matthew D Smith, Qun-Ying Lei, Kun-Liang Guan, Yue Xiong

**Affiliations:** 1 Key Laboratory of Molecular Medicine, Ministry of Education, and Department of Biochemistry and Molecular Biology, Fudan University Shanghai Medical College, Shanghai, China; 2 Molecular and Cell Biology Lab, Institutes of Biomedical Sciences, Fudan University, Shanghai, China; 3 School of Life Sciences, Fudan University, Shanghai, China; 4 Department of Biochemistry and Biophysics, Lineberger Comprehensive Cancer Center, University of North Carolina at Chapel Hill, Chapel Hill, NC, USA; 5 Department of Pharmacology and Moores Cancer Center, University of California at San Diego, La Jolla, CA, USA

## Abstract

Population-based and case–control studies in different ethnicities have linked a polymorphism, C825T, in exon 10 of *GNB3* gene to hypertension and several additional diseases. The 825T allele is associated with alternative splicing and results in a shortened Gβ3 protein, referred to as Gβ3s, which loses 41 amino acids encompassing one WD40 repeat domain. The mechanism of how Gβ3 C825T polymorphism is associated with hypertension has remained unclear, but an impairment of its canonical function in G-protein-coupled receptor signaling has been ruled out. Here, we report that Gβ3, like other Gβ proteins, binds to DDB1 and assembles a DDB1-CUL4A-ROC1 E3 ubiquitin ligase (CRL4A^Gβ3^) to target GRK2 ubiquitination. The loss of the 41 amino-acid residues disrupts the Gβ3-DDB1 binding and impairs the function of Gβ3s to ubiquitinate GRK2. GRK2 ubiquitination levels were decreased and protein levels were accumulated in the blood samples of Gβ3 825T allele carriers. Deletion of *Cul4a* in mice resulted in systolic pressure increased and weakened heart function in male mice that can be partially rescued by the deletion of one *Grk*2 allele. These results reveal a mechanism explaining the link between Gβ3 C825T polymorphism and hypertension.

## Introduction

**Figure 1:**
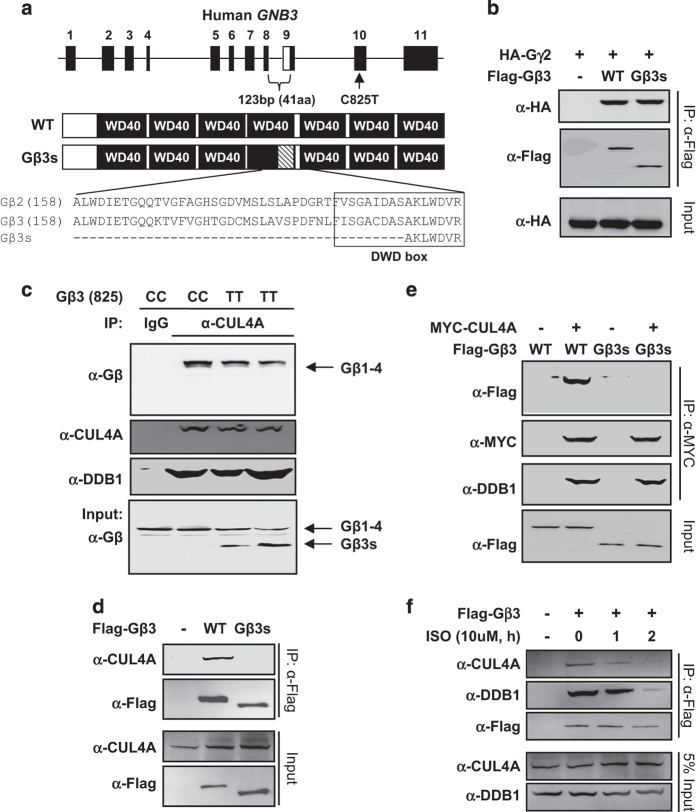
C825T-associated Gβ3s disrupts its binding to CUL4A–DDB1. (**a**) Schematic diagram of genome structure and protein domains of human Gβ3. The C825T is located in the exon 10 and the T allele is associated with splicing variant, Gβ3s, which has deleted 123 bp in exons 8 and 9, and an in-frame deletion of 41 amino-acid residues. (**b**) Both Gβ3 and Gβ3s bind to Gγ2. HEK293 cells were co-transfected with plasmid expressing indicated proteins. Protein–protein binding was determined by co-immunoprecipitation (co-IP). (‘α-Flag’ means anti-Flag antibody, ‘5% input’ means 5% total protein for IP experiments were loaded, the same below). (**c**) CUL4A–DDB1 binds to endogenous Gβ, but not Gβ3s. A measure of 2 ml of total blood samples from different Gβ3 825 allele carried in hypertensive patients was used to immunoprecipitate and was analyzed by immunoblotting. (**d**, **e**) Wild-type Gβ3, but not disease-associated Gβ3s variant, binds to CUL4A. HEK293 cells were transfected with plasmids expressing indicated proteins and protein–protein binding was determined by co-IP. (**f**) Isoproterenol (ISO) decreases CUL4A–DDB1-Gβ3 binding. HEK293 cells were transfected with plasmids expressing Flag-Gβ3 and then treated with ISO (10 μM) for the indicated lengths of time. The levels of individual proteins and the protein–protein interaction was determined by co-IP and immunoblotting using the antibodies indicated.

G-protein-coupled receptors (GPCRs) comprise the largest known family of cell-surface receptors, regulate numerous physiological processes and have a major role in medicine with ~30% of current therapeutics targeting these seven transmembrane receptors [1, 2]. Canonical signals from GPCRs are commonly relayed to intracellular effector proteins by trimeric G proteins, composed of an α, β and γ subunit (Gαβγ) [3]. Impaired GPCR signaling is linked with a wide range of human diseases, including heart disease, cancer and inflammation [4–6]. The G-protein beta 3 subunit (GNB3) C825T polymorphism was detected through a candidate gene approach using cell lines with enhanced G-protein activation from patients with essential hypertension [7]. Independent studies have confirmed an association of the 825 T allele with hypertension in Caucasians [8]. Multiple subsequent population-based and case–control studies in different ethnicities have supported the association between this polymorphism with hypertension, as well as obesity and atherosclerosis, but the mechanism underlying the 825 T polymorphism remains unclear [8–12]. The 825 T allele in exon 10 causes alternative splicing that deletes 123 bp in exon 9, resulting in an in-frame deletion of 41 amino-acid residues and a shortened splice variant of the Gβ3, referred to as Gβ3s [7] (see diagram in Figure 1a). Gβ3s still binds to Gα and Gγ, and also has activity in intracellular signal transduction [3, 7, 8, 10]. Recently, we reported a non-canonical function of Gβ subunits in GPCR signaling that Gβ2 binds to CUL4A–DDB1 and assembles an E3 ubiquitin ligase complex, CRL4^Gβ2^ (the substrate-recruiter DWD protein Gβ is superscripted), to promote the ubiquitination and degradation of GRK2 [13], a member of G-protein-coupled receptor kinases (GRKs, also known as β-adrenergic receptor kinase or β-ARKs) [14].

GRK2 has an important role in the GPCR desensitization process [15], and abnormally elevated GRK2 protein level is linked with multiple pathological conditions in humans [16], including myocardial infarction [17], heart failure and portal hypertension [18]. Gβ2 and other Gβ subunits are WD40 proteins and interact with DDB1 via a mechanism shared with other DDB1-binding WD40 (DWD, also known as DCAF) proteins [19–22], including a conserved DWD box we previously defined [19]. Notably, the 41 residues lost in the Gβ3s variant locate in the fourth WD repeat and contain more than half of the DWD box (Figure 1a). This raises the possibility that the 825 T allele may impair the function of Gβ3s in assembly of CRL4 E3 ligase and targeting GRK2 ubiquitination. This study was directed to test this possibility.

## Results

### C825T-associated Gβ3s disrupts its binding to CUL4A–DDB1

We first confirmed the binding of Gβ3 and Gβ3s with Gγ2 (Figure 1b). We then examined the endogenous interaction of Gβ3 and Gβ3s with different subunits of CRL4 complex in human primary blood samples from different *GNB3* carried hypertensive patients: two 825C homozygous carriers and two 825 T homozygous carriers. Patients were considered to be hypertensive in these experiments based on a systolic blood pressure >140 mm Hg and diastolic pressure >90 mm Hg. We used anti-CUL4A antibody that can co-immunoprecipitate multiple CRL4 components, including both DDB1 and different Gβ subunits [13], and an anti-Pan Gβ antibody that recognizes Gβ3s (Figure 1c) to confirm that Gβ3s was specifically expressed in 825 T carriers, but not in 825C carriers. Notably, Gβ3s was not detected in the CUL4A immunocomplex when other Gβ proteins were readily detected. To confirm this result, we ectopically expressed Flag-tagged Gβ3 and Gβ3s in HEK293 cells and examined their interaction with endogenous CUL4A. Whereas Flag-Gβ3 readily bound with endogenous CUL4A, we found that Flag-Gβ3s did not (Figure 1d). Similar results were obtained using ectopically expressed MYC-tagged CUL4A and Flag-tagged Gβ3/Gβ3s, which showed that Gβ3, but not Gβ3s, bound with CUL4A–DDB1 (Figure 1e). Isoproterenol, a drug used clinically for its inotropic and chronotropic effects on the heart and as a sympathomimetic β-AR agonist, reduces the association of Gβ2 with DDB1–CUL4A [13]. We found that treatment of HEK293 cells with isoproterenol also effectively reduced the association of Gβ3 with CUL4A (Figure 1f), suggesting that Gβ3-DDB1/CUL4A association, such as the Gβ2-DDB1/CUL4A complex, was regulated by GPCR signaling. Together, these results indicate that Gβ3 interacts with DDB1–CUL4A, that this regulation is subjected to the regulation by GPCR signaling and that Gβ3s resulting from disease-linked C825T polymorphism impairs its binding to CUL4A–DDB1.

### Gβ3s loses the function to target GRK2 ubiquitination and stabilizes GRK2

**Figure 2:**
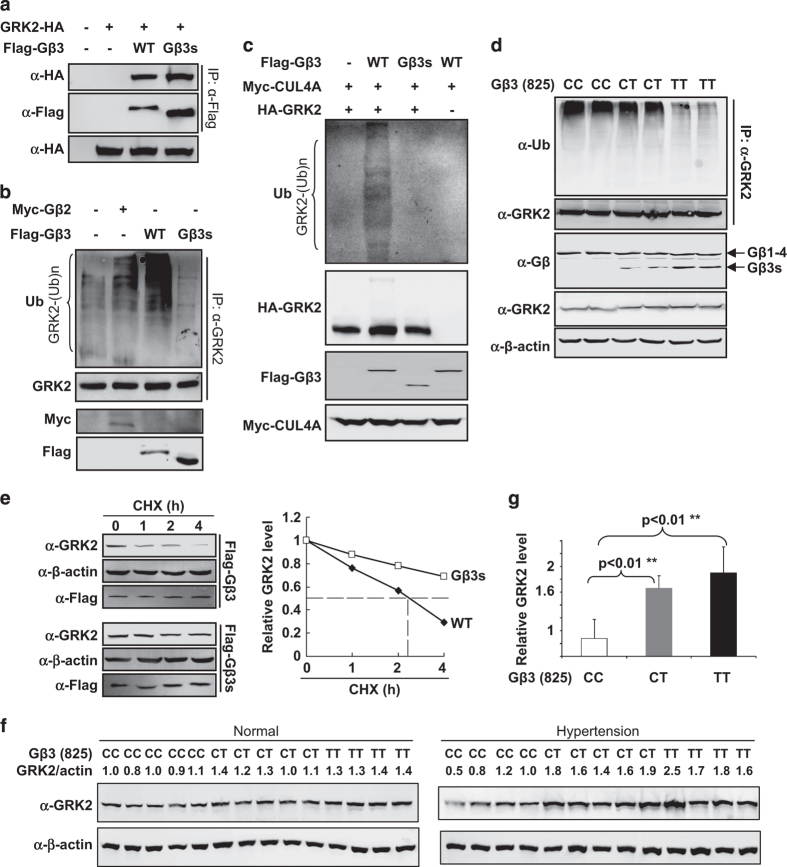
Gβ3s loses the ability to target GRK2 ubiquitination and stabilizes GRK2. (**a**) Both Gβ3 and Gβ3s bind to GRK2. HEK293 cells were transfected with plasmids expressing indicated proteins and protein–protein interaction was determined by co-IP. (**b**) Gβ3, but not Gβ3s, promotes GRK2 ubiquitination. HEK293 cells were transfected with plasmids expressing indicated proteins. Endogenous GRK2 was immunoprecipitated and analyzed for ubiquitination by immunoblotting. (**c**) *In vitro* ubiquitination of GRK2 by CRL4^Gβ3^ E3 ligase, Gβ3s loses the ability to target GRK2 ubiquitination. Purified GRK2 protein was incubated with CUL4A immune complex alone or with purified Gβ3/Gβ3s in the presence of E1, E2, ATP and ubiquitin. After termination, the reaction mixtures were resolved by SDS-polyacrylamide gel electrophoresis, followed by immunoblotting with indicated antibodies. (**d**) Endogenous GRK2 ubiquitination level decreases in *GNB3* 825 T allele samples. A measure of 2 ml of total blood samples from different hypertensive patients who were/were not Gβ3 825 T allele carriers was used to immunoprecipitate and analyzed for ubiquitination by immunoblotting. (**e**) Wild-type Gβ3, but not disease-associated Gβ3s variant, promotes the destabilization of GRK2. HEK293 cells were transfected with plasmids expressing indicated proteins. The half-life of GRK2 protein was determined by CHX treatment for different lengths of time as indicated, followed by immunoblotting and quantification. (**f**) High GRK2 level is associated with Gβ3s C825T polymorphism. A measure of 1 ml total blood samples from different Gβ3 825 allele carriers (left panel) or hypertensive patients (right panel) was used to for western blotting by the antibodies indicated. (**g**) Statistical analysis of GRK2 protein level in different *GNB3* carriers. Comparisons between the two groups were performed with unpaired, two-tailed Student’s *t*-test (Excel software). *P*-values <0.01 were marked by **. Data are presented as the mean±s.d.

The main function of the association of Gβ2 with DDB1–CUL4A is to form the CRL4 E3 ligase complex (CRL4A^Gβ2^) and to ubiquitinate GRK2 [13]. When assayed directly by expression and co-immunoprecipitation, GRK2 was able to bind to both wild-type Gβ3 and Gβ3s (Figure 2a). To determine whether Gβ3 and Gβ3s could regulate the level of GRK2 ubiquitination, Gβ3 or Gβ3s was overexpressed in HEK293 cells, and then the ubiquitination of endogenous GRK2 was determined. The ubiquitination of endogenous GRK2 protein was readily detected and significantly enhanced by the expression of Gβ2 or Gβ3, but not Gβ3s (Figure 2b). We noted that ubiquitinated GRK2 levels in cells expressing Gβ3s were even lower than untransfected cells, suggesting a dominant-negative inhibition of endogenous Gβ3 by the CUL4A/DDB1-binding-deficient Gβ3s. An *in vitro* ubiquitination assay showed that incubation of immunopurified GRK2 with immunopurified CUL4A and Gβ3 complexes resulted in robust GRK2 ubiquitination in the presence of E1, E2, ATP and ubiquitin (Figure 2c). GRK2 ubiquitination bands were not observed in the absence of Gβ3 (lane 1) or the substrate GRK2 (lane 4), as they were only detected in the presence of Gβ3 (lane 2), but not Gβ3s (lane 3). Together, these *in vivo* and *in vitro* ubiquitination assays demonstrate that Gβ3, like Gβ2, also targets GRK2 for ubiquitination and that this activity is disrupted in Gβ3s.

Endogenous GRK2 ubiquitination levels in human blood samples from individuals with *GNB3* 825C or 825 T allele were also examined. Anti-GRK2 beads were used to pull-down endogenous GRK2 protein of blood samples from patients with hypertension, and an anti-ubiquitin antibody was used to detect the endogenous GRK2 ubiquitination level. GRK2 ubiquitination levels were significantly decreased in *GNB3* 825 T allele samples when compared with those from samples with 825C homozygous or 825C/T heterozygous alleles (Figure 2d).

We next examined whether Gβ3s regulates the stability of GRK2 protein and found that GRK2 is a relatively unstable protein with an estimated half-life (*t*_1/2_) of ~2.3 h in cells expressing wild-type Gβ3 (Figure 2e). Ectopic expression of Gβ3s significantly increased the half-life of GRK2 beyond the experimental duration (4 h; Figure 2e). This suggests that overexpression of Gβ3s stabilizes GRK2 protein, a notion that is consistent with the finding that ectopic expression of Gβ3s also reduces the ubiquitination of endogenous GRK2 (Figure 2b).

The protein levels of endogenous GRK2 were determined in blood samples from both normal and hypertensive patients. We found that in normal patients, homozygous *GNB3* 825 T was associated with higher levels of GRK2 compared with homozygous 825C or heterozygous 825C/T (Figure 2f, left panel). Likewise, GRK2 was also accumulated in *GNB3* 825 T homozygote samples from hypertensive patients (Figure 2f, right panel). The differences in GRK2 protein steady-state levels between possessing the CC, CT or TT genotype were significant (*P*<0.01; Figure 2g). These results further support the notion that Gβ3 C825T polymorphism is associated with increased GRK2 protein level and that the increase is not the consequence of, but may be exacerbated during hypertension.

### Male *Cul4a*^*−/−*^ mice develop hypertension that is partially rescued by loss of one *Grk2* allele

**Figure 3:**
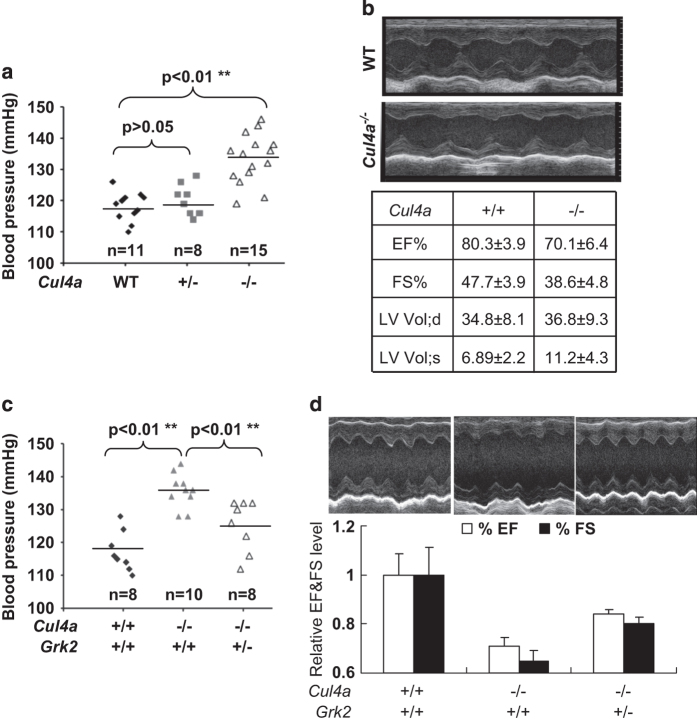
Male *Cul4a*^*−/−*^ mice develop hypertension that is partially rescued by loss of one Grk2 allele. (**a**) Male *Cul4a*^*−/−*^ mice develop hypertension. The systolic blood pressure (SBP) of 34 age-matched male mice of different genotypes was determined by NIBP mouse blood pressure meter machine. The statistical significances of heart weight differences between different genotypes were determined by *P*-value calculation as indicated. (**b**) *Cul4a*^*−/−*^ male mice have weaker heart function. The heart function of six pairs of littermate wild-type and *Cul4a*^*−/−*^ male mice were determined by echocardiogram (ECHO) analysis. The values of heart ejection fraction (EF %), fractional shortening (FS %), end-diastolic (LV vol; d) and end-systolic (LV vol; s) of left ventricular were the average of six mice of each genotype. (**c**) Loss of one Grk2 allele partially rescues hypertension of *Cul4a*^*−/−*^ mice. The SBP of 26 age-matched male mice of different genotypes was determined. The statistical significance of heart weight differences between different genotypes was indicated by the *P-*value shown. (**d**) Loss of one Grk2 allele partially rescues weak heart function of *Cul4a*^*−/−*^ mice. The heart function for different genotype male mice was determined by ECHO analysis (under isoflurane treatment). The values of heart ejection fraction (EF %) and fractional shortening (FS %) were compared with wild-type mice.

The findings that both Gβ2 and Gβ3 target GRK2 ubiquitination led us to seek genetic and physiological evidences supporting the function of CUL4A–DDB1-Gβ E3 ligase in the regulation of GRK2 *in vivo*. We previously characterized *Cul4a* null mice and found that, although overall appearing to development normally, the male, but not female *Cul4a*^*−/−*^ mice develop cardiac hypertrophy [13]. Further analyses of blood pressures and cardiac functions were carried out, and the systolic blood pressures of 34 age-matched, 10-week-old male mice (11 wild-type mice, 8 *Cul4a*^+/-^ mice and 15 *Cul4a*^*−/−*^ mice) were determined. Compared with wild-type mice, the systolic blood pressure of *Cul4a*^*−/−*^ male mice was significantly higher (*P*<0.01; Figure 3a). To determine whether the hypertension associated with *Cul4a* deletion results in cardiac dysfunction, two-dimensional echocardiogram analysis was performed for male *Cul4a*^*−/−*^ and wild-type mice. *Cul4a*^*−/−*^ male mice displayed multiple parameters of cardiac dysfunction compared with wild types, including increased left ventricular (LV) volume (6.89 vs 11.2 μl end-systolic volume in wild-type vs *Cul4a*^*−/−*^) and decreased fractional shortening (38.6±4.8% vs 47.7±3.9% in wild type vs *Cul4a*^*−/−*^; Figure 3b).

The cardiac hypertrophy defect seen in *Cul4a*^*−/−*^ mice can be partially rescued by deletion of one allele of *Grk2* [13]. To demonstrate genetically that Grk2 mediates the function of Cul4a in maintaining blood pressure homeostasis and cardioprotection, we characterized blood pressure and cardiac function of *Cul4*; *Grk2* double-mutant mice. Systolic blood pressure of *Cul4a*^*−/−*^; *Grk2*^*+/−*^ male mice was partially decreased compared with *Cul4a*^*−/−*^ mice (Figure 3c). Echocardiogram analysis showed that although the *Cul4a*^*−/−*^ male mice had weakened cardiac function (~70% ejection fraction and 65% fractional shortening compared with wild type), deletion of one *Grk2* allele partially restored the heart function in *Cul4a*^*−/−*^; *Grk2*^*+/−*^ male mice (~120% ejection fraction and 125% fractional shortening; Figure 3d). Together, these molecular, cellular and physiological analyses establish that Cul4A-Gβ-mediated GRK2 degradation has a direct and important role in the regulation of heart function in male mice and that an impairment of this function, such as those seen in C825 T carriers, may contribute to the development of hypertension and weakened heart function.

## Discussion

In 1998, Gβ3 C825T polymorphism was first reported to be associated with hypertension and was subsequently confirmed by multiple population-based or case–control studies in different ethnicities. It has since been extended to diseases directly linked with hypertension such as atherosclerosis, left ventricular hypertrophy, stroke and myocardial infarction, as well as additional diseases such as obesity, insulin resistance and depression [7, 8, 10–12, 23, 24]. The molecular mechanism underlying the change in Gβ3 protein caused by C825T polymorphism, however, has remained unclear [25]. Deletion of 41 amino-acid residues resulting from the 825T allele does not significantly impact the binding of Gβ3s to Gα and Gγ, nor the function for GDP/GTP exchange [7, 10], leading to the notion that the truncated Gβ3s remains biologically active, at least as a signal transducer for GPCR. Although one study reported that Gβ3s is unstable and fails to bind with either Gγ or Gα [26], we found that Gβ3s appears to bind with Gγ3 indistinguishable from that of wild-type Gβ3 (Figure 1b). Instead, we found that loss of the 41 residues abolished Gβ3s binding to DDB1. We demonstrated that both ectopically and endogenously expressed Gβ3s lost its ability to interact with DDB1 and the ability to assemble an active CRL4 E3 ligase complex. Supporting the disruptive effect on association with DDB1 and formation of CRL4 E3 complex by the deletion of 41 residues, we show that Gβ3s, although still capable of binding to substrate GRK2, fails to catalyze GRK2 ubiquitination and promotes GRK2 stabilization and accumulation in cells. The pathological significance of an impairment of GRK2 degradation by the CRL4A^Gβ^ E3 ligases, including CRL4A^Gβ3^, is supported by the finding that *Cul4a* mutant mice accumulate higher levels of Grk2 protein and develop cardiac hypertrophy [26], hypertension and weakened heart function that can be partially rescued by the deletion of Grk2 (Figure 3). Although CUL4A can assemble potentially multiple E3 ligases including several CRL4A^Gβ^ complexes, we suggest that loss of the ability to assemble CRL4A^Gβ3^ complex and target GRK2 ubiquitination represents a major mechanism underlying the disease-associated C825T polymorphism.

## Materials and Methods

### Cell culture and transfection

HEK293 and HEK293T cells were cultured in Dulbecco’s modified Eagle’s medium supplemented with 10% newborn calf serum, 100 U ml^−1^ penicillin and streptomycin (Gibco, Grand Island, NY, USA). Mouse embryonic fibroblast cells were maintained in Dulbecco’s modified Eagle’s medium supplemented with 10% fetal calf serum (Gibco), 1% L-glutamine, 100 U ml^−1^ penicillin and streptomycin. Cell transfection was performed using Lipofectamine 2000 (Life Technologies, Grand Island, NY, USA) or calcium phosphate method. Cells were collected at 36–48 h post transfection for protein analyses.

### Antibodies and immunological procedures

Protein lysates were prepared by lysing HEK293 cells in a buffer containing 50 mM Tris-HCl (pH 7.5), 150 mM NaCl, 0.1% SDS, 0.5% deoxycholate, 0.5% Nonidet P-40, 1 mM EDTA, 1 mM PMSF, 25 mM NaF and a mixture of protease inhibitors. Cell lysate (20 μg) was resolved by SDS-polyacrylamide gel electrophoresis, followed by western blotting analysis. Antibodies recognizing Flag (Sigma, St Louis, MO, USA), GRK2 (Santa Cruz, Dallas, TX, USA), HA (Santa Cruz), Myc (Santa Cruz), pan-Gβ antibody (Santa Cruz) and β-actin (Cell Signaling, Shanghai, China) were purchased commercially. Antibodies to DDB1 and CUL4A have been described previously [27].

For immunoprecipitation experiments, 800 μg of total protein in cell lysate was incubated with anti-Flag M2-agarose (Sigma) or anti-GRK2 beads (Santa Cruz) for 3 h at 4 °C. Beads were washed three times with lysis buffer and centrifuged at 2 000 *g* for 3 min between each wash. Protein was eluted from beads with 50 μl of SDS sample buffer. Lysates were resolved on 8–15% SDS-polyacrylamide gel electrophoresis gels and transferred on to nitrocellulose (Bio-Rad, Hercules, CA, USA) for western blotting.

For experiments involving human blood, 2–5 ml of total blood samples was used for each immunoprecipitation experiment. The blood samples were centrifuged at 2 000 *g* for 5 min, and the precipitated blood cells were lysed in a buffer containing 50 mM Tris-HCl (pH 7.5), 150 mM NaCl, 0.1% SDS, 0.5% deoxycholate, 0.5% Nonidet P-40, 1 mM EDTA, 1 mM PMSF, 25 mM NaF and a mixture of protease inhibitors. The total protein from cell lysates was used for immunoprecipitation experiments, which were described above.

### *In vitro* ubiquitin ligation assays

Plasmids expressing Myc-CUL4A, HA-GRK2, Flag-Gβ3 or Flag-Gβ3s were individually transfected into 293 T cells by Lipofectamine 2000. At 48 h after transfection, cells were lysated into a buffer containing 50 mM Tris-HCl (pH 7.5), 150 mM NaCl, 0.1% SDS and a cocktail of protease inhibitors, followed by immunoprecipitation using Myc or HA sepharose (Santa Cruz). Immunocomplexes were washed with the lysis buffer and eluted by Myc or HA antigen peptides. Immunopurified HA-GRK2 protein was mixed with Myc-CUL4A and Myc3-Gβ2 in a ubiquitin ligation buffer (50 mM Tris-HCl/pH 7.4, 5 mM MgCl_2_, 2 mM NaF, 2 mM ATP, 10 nM okadaic acid, 0.6 mM dithiothreitol, 12 μg of bovine ubiquitin, 1 μg of ubiquitin (Sigma), 60 ng of E1 (E301, Boston Biochem, Cambridge, MA, USA), 500 ng of E2 (human Ubc5c), final volume=30 μl). The reaction was incubated at 37 °C for 1 h on a rotator with slow shaking and then terminated by boiling at 95 °C with SDS sample buffer for 10 min before SDS-polyacrylamide gel electrophoresis. GRK2 ubiquitination was examined by immunoblotting with either anti-ub or anti-HA antibody.

### Noninvasive mouse blood pressure measurement

The blood pressure of 2-month-old male mice was measured using tail cuff plethysmography blood pressure systems (IITC Life Science, Woodland Hills, CA, USA). Six measurements were collected on each mouse, and the average was then calculated after excluding the lowest and highest readings.

### Echocardiographic analysis

Cross-sectional, two-dimensional and Doppler transthoracic echocardiography was performed by experienced sonographers using a Vevo770 Imaging System (VisualSonics Inc, Toronto, ON, Canada). The chests of the male mice were shaved and treated with a chemical hair remover to reduce ultrasound attenuation. Heart rate and core temperature were continuously monitored. Mice were anesthetized with 1–2% isofluorane. Images were stored in the Visual Sonics Imaging System for offline analysis. The values of heart ejection fraction (EF %), fractional shortening (FS %), end-diastolic (LV vol; d) and end-systolic (LV vol; s) of left ventricular were the average of six mice of each genotype.

### Statistical analysis

Comparisons between the two groups were performed with unpaired, two-tailed Student’s *t*-test (Excel software, Microsoft Corp., Shanghai, China). *P*-values <0.05 were considered statistically significant. Data are presented as the mean±s.d.
